# The Impact of Geography in Hepatocellular Carcinoma: A Retrospective Population Based Study

**DOI:** 10.3390/curroncol28010042

**Published:** 2021-01-12

**Authors:** Irene S. Yu, Shiru L. Liu, Valeriya Zaborska, Tyler Raycraft, Sharlene Gill, Howard Lim, Janine M. Davies

**Affiliations:** 1 Department of Medical Oncology, BC Cancer Vancouver, Vancouver, BC V5Z 4E6, Canada; ireneyu@alumni.ubc.ca (I.S.Y.); sgill@bccancer.bc.ca (S.G.); hlim@bccancer.bc.ca (H.L.); 2 Faculty of Medicine, University of British Columbia, Vancouver, BC V6T 1Z3, Canada; vzaborska@alumni.ubc.ca (V.Z.); tyler.raycraft@alumni.ubc.ca (T.R.); 3 Department of Medical Oncology, BC Cancer Fraser Valley, Surrey, BC V3V 1Z2, Canada; lucy.liu@bccancer.bc.ca

**Keywords:** hepatocellular carcinoma, HCC, urban, rural, disparity

## Abstract

Background: The treatment of hepatocellular carcinoma (HCC) includes different therapeutic modalities and multidisciplinary tumor board reviews. The impact of geography and treatment center type (quaternary vs. non-quaternary) on access to care is unclear. Methods: A retrospective chart review was performed on HCC patients who received sorafenib in British Columbia from 2008 to 2016. Patients were grouped by Statistics Canada population center (PC) size criteria: large PC (LPC), medium PC (MPC), and small PC (SPC). Access to specialists, receipt of liver-directed therapies, and survival outcomes were compared between the groups. Results: Of 286 patients, the geographical distribution was: LPC: 75%; MPC: 16%; and SPC: 9%. A higher proportion of Asians (51% vs. 9% vs. 4%; *p* < 0.001), Child–Pugh A (94% vs. 83% vs. 80%; *p* = 0.022), and hepatitis B (37% vs. 15% vs. 4%; *p* < 0.001) was observed in LPC vs. MPC vs. SPC, respectively. LPC patients were more likely referred to a hepatologist (62% vs. 48% vs. 40%; *p* = 0.031) and undergo transarterial chemoembolization (TACE) (43% vs. 24% vs. 24%; *p* = 0.018). Sixty percent were treated at a quaternary center, and the median overall survival (OS) was higher for patients treated at a quaternary vs. non-quaternary center (28.0 vs. 14.6 months, respectively; *p* < 0.001) but similar when compared by PC size. Treatment at a quaternary center predicted an improved survival on multivariate analysis (hazard ratio (HR): 0.652; 95% confidence interval (CI): 0.503–0.844; *p* = 0.001). Conclusions: Geography did not appear to impact OS but patients from LPC were more likely to be referred to hepatology and undergo TACE. Treatment at a quaternary center was associated with an improved survival.

## 1. Background

An estimated 3000 new cases of liver cancer are diagnosed in Canada annually, and there are 1400 projected deaths per year [1]. Hepatocellular carcinoma (HCC) makes up the majority of liver cancers, which carry a poor prognosis with five-year survival estimated at 19%, and mortality rates have increased by 2–3% per year in the past three decades [1]. Treatment intent is palliative in approximately 45% of cases who present with regional or distant metastases at diagnosis [2].

Historically, systemic options have been limited, and local therapies are the mainstay of treatment in HCC. Local strategies include surgery, radiation, and interventional radiology treatments; however, there are limited data and no clear guidelines to determine the best sequence of care or to select the optimal intervention. Multidisciplinary conferences and the involvement of multiple specialist clinicians are associated with reduced mortality [3] but may not be available in all geographic locations.

In Canada, universal healthcare provides an ease of financial access to care; however, given the vast geography, there may be disparities between urban and rural locations. A large cross-sectional Canadian study showed that those from a major urban core were more likely to consult a specialist physician (odds ratio (OR): 1.24) [4]. Overall, HCC is a unique disease entity for which a multidisciplinary approach is optimal given the complexity of treatment options. This study evaluated the impact of geography on access to different specialists and liver-directed treatment options, as well as exploring associations with survival outcomes. The objective was to identify any disparities in access to care for HCC patients, providing information for clinicians and health systems to work together to bridge any gaps.

## 2. Methods

### 2.1. Description of Study Population

British Columbia (BC) Cancer operates six regional cancer centers that provide oncology services including chemotherapy, radiation therapy, and supportive care to the residents of BC, Canada, which has an estimated population of 4,648,055 [5]. We included patients with HCC, 18 years of age or older, who were dispensed at least one cycle of sorafenib from a BC Cancer pharmacy between January 2008 and December 2016. There is no centralized database of HCC patients in our province because HCC can be a radiographic and/or pathologic diagnosis; thus, our database was established on the receipt of sorafenib in any setting. Patients were identified through the BC Cancer Pharmacy database, which dispenses publicly-funded anti-cancer therapy for the province. Surgical and interventional procedures are performed at several acute care hospitals in BC.

### 2.2. Definitions of Population Center Size/Geography

Patients’ postal codes based on their home address were correlated with the corresponding population center (PC), then categorized to one of three Statistics Canada population center size groups [6]: small (populations between 1000 and 29,999), medium (between 30,000 and 99,999), and large (100,000 or more) population centers (SPC, MPC, and LPC, respectively).

### 2.3. Description of Treatment Centers

Treatment location was classified as quaternary versus non-quaternary based on the location of the treating medical oncologist. During the time frame of the study, only one regional cancer center was classified as quaternary, with all comprehensive services available including hepatobiliary surgery, interventional radiology, hepatology, and weekly multidisciplinary liver tumor board (MDTB) meetings held to discuss cases and reach group consensus recommendations. The other five non-quaternary regional cancer centers had most but not all of these services. All centers had radiation oncology, medical oncology, internal medicine, gastroenterology, and general surgery services available; some centers had multidisciplinary rounds ranging from weekly to monthly. All treatment centers were located in an LPC except one (MPC).

### 2.4. Data Collection and Statistical Analyses

Patient demographics, cancer, and treatment information were collected through a retrospective chart review. Specific access outcomes of interest included the receipt of subspecialist consultations and local liver-directed therapies. Univariate analyses used Pearson’s chi-square test and maximum likelihood ratio chi-square test. Multivariate analysis for predictors of overall survival (OS) was conducted using a Cox regression analysis, employing a forward likelihood ratio method after satisfying the proportional hazards assumption. Variables were entered into the model if *p* < 0.05 and removed from the model if *p* > 0.10. Variables assessed for the final model included age, gender, race, Eastern Cooperative Oncology Group (ECOG) performance status, Child–Pugh score, alpha-fetoprotein (AFP) level, metastatic disease at diagnosis, PC size, and quaternary versus non-quaternary treatment center. OS was evaluated from time of initial radiographic HCC diagnosis to death from any cause, calculated by the Kaplan–Meier function using a two-sided alpha and a threshold of 0.05 for statistical significance. All analyses were performed using IBM SPSS (Version 26).

### 2.5. Ethics Approval

The research study was approved by the University of British Columbia/BC Cancer Agency Research Ethics Board (H17-01147).

## 3. Results

A total of 286 patients were identified and included for analysis (Table 1). The group variables were a median age of 62 years, 81.8% male, 39.9% Asian, 82.4% ECOG 0/1, and 90.5% Child–Pugh A. In terms of liver disease etiology, 30.8% had hepatitis B, 32.2% had hepatitis C, and 25.5% had documented alcohol-related cirrhosis. Distribution by PC size included 75.2% from LPC, 16.1% from MPC, and 8.7% from SPC. When comparing by PC size, groups were balanced by age, gender, performance status, Barcelona Clinic Liver Cancer (BCLC) staging, and AFP level (≥400 μg/L vs. < 400 μg/L). There was a higher proportion of patients who were Asian (50.7% vs. 8.7% vs. 4.0%; *p* < 0.001), Child–Pugh A status (93.5% vs. 82.6% vs. 80.0%; *p* = 0.022), and hepatitis B (HBV) etiology (37.2% vs. 15.2% vs. 4.0%; *p* < 0.001) in the LPC group compared to the MPC and SPC groups, respectively. Alcohol-related liver disease was less frequent in LPC patients (20.9%) and more common in MPC and SPC patients (37.0% and 44.0%, respectively; *p* = 0.007). There was no significant difference in the proportion of patients with metastatic disease at diagnosis between the LPC, MPC, and SPC groups.

When evaluated by whether the patient’s treatment center was at a quaternary center versus non-quaternary center, 59.8% (171/286) of patients were treated at the quaternary center, which averaged 19 new patients per year initiated on sorafenib. The non-quaternary sites averaged less than five new patients per year. Of patients who were treated at a quaternary center, 85.4%, 9.9%, and 4.7% of patients were from LPC, MPC, and SPC, respectively.

Patients from LPC were more likely to see a hepatologist (62.3% vs. 47.8% vs. 40.0%; *p* = 0.031) compared to MPC and SPC, respectively (Table 2). Otherwise, referrals to gastroenterology, internal medicine, hepatobiliary surgery, general surgery, and interventional radiology were not significantly different across the LPC, MPC, and SPC groups. Regarding accessibility to surgical services, 51.0% patients were assessed by a hepatobiliary surgeon, and 15.0% were assessed by a general surgeon. One-quarter (24.1%) of patients were assessed by an interventional radiologist. For medical consultants, 58.0% of patients were referred to a hepatologist, 29.0% were referred to a gastroenterologist, and 18.5% were referred to a general internist.

The most common local therapies used at some point in the patient’s treatment trajectory (Table 3) were transarterial chemoembolization (TACE; 38.1%), resection (27.3%), and ablation (15.0%). For those who received TACE, median number of TACE procedures was 2 (range of 1–9). Other local therapies were used less commonly, including alcohol injection (4.5%), transarterial embolization (TAE; 3.8%), transarterial radioembolization with Yttrium-90 (TARE; 3.8%), liver transplant (3.1%), and stereotactic radiation (1.0%). Access to different therapies were similar across population centers, except those from LPC were more likely to have undergone TACE versus MPC and SPC (42.8% vs. 23.9% vs. 24.0%; *p* = 0.018). Those from LPC were more likely to have received any liver-directed therapy in the course of their treatment (63.7% LPC vs. 43.5% MPC and 48.0% SPC; *p* = 0.020).

**Figure 1:**
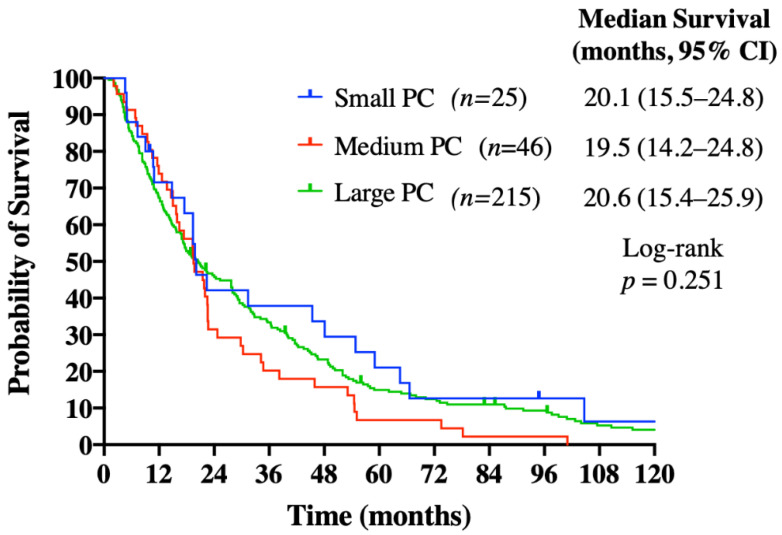
Overall survival analysis calculated from HCC diagnosis, by population center size. Abbreviations: PC: population center.

**Figure 2:**
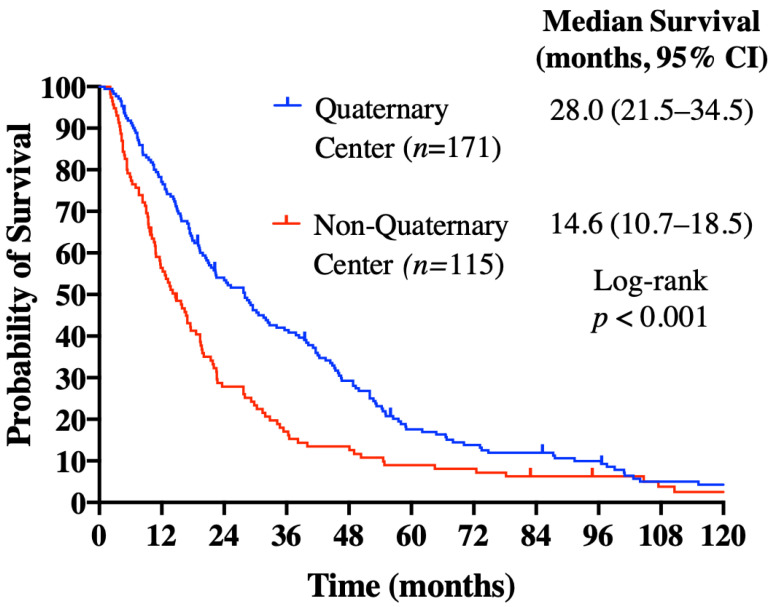
Overall survival analysis calculated from HCC diagnosis, by quaternary versus non-quaternary treatment center.

The median OS (mOS) calculated from HCC diagnosis was 19.9 months for the study population, and it was similar between patients from SPC, MPC, and LPC at 20.1, 19.5, and 20.6 months, respectively (*p* = 0.251) (Figure 1). By quaternary versus non-quaternary center, patients treated at a quaternary center had a longer mOS of 28.0 months versus 14.6 months at a non-quaternary site (*p* < 0.001) (Figure 2). Quaternary center status remained an independent predictor of survival on multivariate analysis (hazard ratio (HR): 0.652; 95% confidence interval (CI): 0.503–0.844; *p* = 0.001), as did an ECOG performance status of 0/1 (HR: 0.417; 95% CI: 0.300–0.579; *p* < 0.001) and AFP level <400 μg/L (HR: 0.679; 95% CI: 0.527–0.873; *p* = 0.003) (Table 4). Forty-two (14.7%) patients went on to receive further treatment with systemic therapy, local therapy, and/or clinical trials, with no difference observed between the PC groups (15.8% LPC vs. 8.7% MPC vs. 16.0% SPC; *p* = 0.416). Of these patients, fifteen had systemic therapy post sorafenib, including 13 who received doxorubicin; 21 received liver directed therapies and/or radiation to extrahepatic sites.

## 4. Discussion

Though there is universal healthcare coverage in Canada, this may not necessarily translate into universal access to care due to geographical differences. Three-quarters of our study population lived in an LPC, which is similar to the estimated proportion of the Canadian population living in a census metropolitan area in 2018 at 72% [7].

Referrals to medical and surgical specialists were similar between the PC groups, except that patients from larger centers were more likely to see a hepatologist. This is likely explained by the increased availability of hepatologists in urban settings and their imperative role in the management of underlying liver disease. Referrals to hepatobiliary surgery and interventional radiology usually occur after MDTB meetings, which may explain the lower referral rates to these specialties seen in our study. Both National Comprehensive Cancer Network and American Association for the Study of Liver Diseases guidelines support MDTB meetings in the treatment of HCC [8,9], which is associated with improved survival outcomes across different countries [3,10,11,12]. In practice, this is generally limited to academic institutions in urban areas where there are more specialists available to attend. The model in BC, in which providers from any affiliated cancer center can access the provincial MDTB, may be a way to overcome geographical barriers. The increased adoption of virtual meetings during the coronavirus disease 2019 pandemic [13] may serve as a potential solution to increase MDTB attendance and access to different consultants, which can also be an opportunity for clinical trial recruitment [14].

Access to local therapies were generally balanced between the different PC groups, with TACE and resection most commonly used. TACE was the only intervention that was more frequently used in patients from larger centers, at nearly double the rate (42.8% for LPC vs. 23.9% for SPC). This illustrates the need for patient cases to be presented for MDTB review and referred for potential TACE if appropriate. Since the study time frame, there has been increased availability of TACE in other BC centers, so we would expect this difference to decrease if it is related to geographic availability. It is possible that no difference was observed for the other local therapies because less than 5% received such therapies. TARE was implemented provincially in 2011, and it was restricted to selected cases including those with portal vein invasion/thrombosis or with the intent of downstaging as a bridge to liver transplant or resection.

Survival outcomes in this study were similar when analyzed by PC size, reflecting that urban versus rural status did not influence patient outcomes. In a study performed by Sun et al. in a Chinese population, a significant decline in liver cancer mortality was seen in the urban population from 1991 to 2014 (average annual percent change: from −1.1% to −1.4%), whereas the mortality rate was stable in the rural population [15]. In the Canadian context, Xu et al. found that patients with biliary cancer who lived more than two hours away from the nearest cancer center were less likely to receive chemotherapy (OR: 0.51; 95% CI: 0.29–0.88) and had decreased survival (HR: 1.27; 95% CI: 1.17–1.37) [16]. This may not be directly translatable to HCC due to differing tumor biology and treatments. Furthermore, biliary cancers may not be consistently reviewed by MDTBs. A retrospective Surveillance, Epidemiology, and End Results database study showed that patients from rural US regions had more advanced stage HCC at diagnosis (OR: 1.10; 95% CI: 1.00–1.20) and higher mortality (HR: 1.05; 95% CI: 1.01–1.08) [17]. Differences in BC’s population-based treatment paradigm, healthcare infrastructure, and practice patterns may have driven our discordant results.

Survival outcomes were improved for patients treated at a quaternary center after adjusting for other factors on multivariate analysis. This is likely multifactorial because quaternary centers have more resources including specialists, interventions, and MDTBs [18]. There may be an element of referral bias; those who undergo certain local therapies such as TACE have earlier disease, and out-of-catchment surgical resection and liver transplant patients may be more likely to get ongoing care at the same center. The difference in survival is not likely impacted by access to second-line treatment after sorafenib, as the numbers were low and similar across the groups. In most cases, the systemic therapy used in the second-line setting was doxorubicin, which historically has shown a three week survival benefit [19] and is not expected to significantly change survival. The study period predated the approval of currently available second-line systemic therapies.

There were several limitations in our study. It is challenging to capture the true incidence of HCC because it is diagnosed both pathologically and radiographically; further studies may look at databases with diagnostic codes in order to capture a more complete population. The sample size was limited because neither earlier stage patients not requiring sorafenib nor those too unwell to receive it were included. Our database also did not capture those who transitioned from one center for their liver-directed therapy to another for medical oncology, although, in practice, treatment tends to continue at the same center. Lastly, the treatment landscape of unresectable HCC has significantly changed over the past several years with multiple advances, including immunotherapy, targeted therapies, and ongoing clinical trials. Whether geography or type of treatment center impacts uptake of these treatments remains unknown.

## 5. Conclusions

In our provincial cohort of HCC patients who received sorafenib, urban versus rural status did not appear to impact referrals to specialists and local therapies, with the exception of referrals to hepatology and receipt of TACE. Patients may benefit from multidisciplinary tumor board reviews to expand the use of TACE and other local therapies with low uptakes. Survival outcomes are not impacted by patients’ geographical location, but those referred to a quaternary center have an associated improved survival.

## Figures and Tables

**Table 1:** Baseline patient characteristics compared by population center size.

Patient Characteristics ^1^		Small Urban Population Center ^2^ (*n* = 25), %	Medium Urban Population Center (*n* = 46), %	Large Urban Population Center (*n* = 215), %	*p*-Value
Age	<65	15 (60.0)	24 (52.2)	109 (50.7)	0.677
	≥65	10 (40.0)	22 (47.8)	106 (49.3)	
Gender	Male	20 (80.0)	37 (80.4)	177 (82.3)	0.928
	Female	5 (20.0)	9 (19.6)	38 (17.7)	
Race	Non-Asian	24 (96.0)	42 (91.3)	106 (49.3)	<0.001
	Asian	1 (4.0)	4 (8.7)	109 (50.7)	
ECOG Performance Status	0–1	21 (84.0)	37 (82.2)	176 (82.2)	0.975
	2–3	4 (16.0)	8 (17.8)	38 (17.8)	
Child–Pugh Score	A	20 (80.0)	3 (82.6)	200 (93.5)	0.022
	B	5 (20.0)	8 (17.4)	14 (6.5)	
BCLC Staging at initiation of sorafenib ^3^	A	0 (0)	0 (0)	2 (0.9)	0.978
	B	1 (4.0)	4 (8.7)	14 (6.5)	
	C	23 (92.0)	41 (89.1)	191 (88.8)	
	D	1 (4.0)	1 (2.2)	6 (2.8)	
Alpha-fetoprotein (µg/L)	<400	11 (47.8)	22 (51.2)	106 (52.5)	0.910
	≥400	12 (52.2)	21 (48.8)	96 (47.5)	
Liver Disease Etiology	Hepatitis B	1 (4.0)	7 (15.2)	80 (37.2)	<0.001
	Hepatitis C	11 (44.0)	18 (39.1)	63 (29.3)	0.180
	Alcohol-associated	11 (44.0)	17 (37.0)	45 (20.9)	0.007
Metastatic Disease at Diagnosis	Yes	15 (60.0)	20 (43.5)	84 (39.1)	0.128
	No	10 (40.0)	26 (56.5)	131 (60.9)	

Abbreviations: ECOG: Eastern Cooperative Oncology Group; BCLC: Barcelona Clinic Liver Cancer. ^1^ Characteristics are at the time of sorafenib initiation, unless otherwise specified. ^2^ One patient included was from an area with a population < 1000. ^3^ Two patients were missing BCLC staging information.

**Table 2:** Patterns of referrals to different medical and surgical consultants during the course of hepatocellular carcinoma (HCC) treatment, by population center size.

Medical or Surgical Specialty Consulted during Course of HCC Treatment		Small Urban Population Center (*n* = 25), %	Medium Urban Population Center (*n* = 46), %	Large Urban Population Center (*n* = 215), %	*p*-Value
Hepatology	Yes	10 (40.0)	22 (47.8)	134 (62.3)	0.031
	No	15 (60.0)	24 (52.2)	81 (37.7)	
Gastroenterology	Yes	7 (28.0)	9 (19.6)	67 (31.2)	0.288
	No	18 (72.0)	37 (80.4)	148 (68.8)	
Internal medicine	Yes	7 (28.0)	12 (26.1)	34 (15.8)	0.134
	No	18 (72.0)	34 (73.9)	181 (84.2)	
Hepatobiliary surgery	Yes	14 (56.0)	20 (43.5)	112 (52.1)	0.498
	No	11 (44.0)	26 (56.5)	103 (47.9)	
General surgery	Yes	5 (20.0)	10 (21.7)	28 (13.0)	0.271
	No	20 (80.0)	36 (78.3)	187 (87.0)	
Interventional radiology	Yes	5 (20.0)	8 (17.4)	56 (26.0)	0.405
	No	20 (80.0)	38 (82.6)	159 (74.0)	

Abbreviations: HCC: hepatocellular carcinoma.

**Table 3:** Receipt of liver-directed therapies during course of HCC treatment, by population center size.

Liver-Directed Therapies during Course of HCC Treatment		Small Urban Population Center (*n* = 25), %	Medium Urban Population Center (*n* = 46), %	Large Urban Population Center (*n* = 215), %	*p*-Value
Liver resection	Yes	8 (32.0)	11 (23.9)	59 (27.4)	0.761
	No	17 (68.0)	35 (76.1)	156 (72.6)	
Liver transplant	Yes	2 (8.0)	0 (0.0)	7 (3.3)	0.115
	No	23 (92.0)	46 (100.0)	208 (96.7)	
Liver ablation	Yes	3 (12.0)	6 (13.0)	34 (15.8)	0.802
	No	22 (88.0)	40 (87.0)	181 (84.2)	
Alcohol injection	Yes	0 (0.0)	3 (6.5)	10 (4.7)	0.259
	No	25 (100.0)	43 (93.5)	205 (95.3)	
Transarterial embolization	Yes	0 (0.0)	1 (2.2)	10 (4.7)	0.256
	No	25 (100.0)	45 (97.8)	205 (95.3)	
Transarterial chemoembolization	Yes	6 (24.0)	11 (23.9)	92 (42.8)	0.018
	No	19 (76.0)	35 (76.1)	123 (57.2)	
Radioembolization	Yes	1 (4.0)	0 (0.0)	10 (4.7)	0.138
	No	24 (96.0)	46 (100.0)	205 (95.3)	
Stereotactic radiation	Yes	0 (0.0)	0 (0.0)	3 (1.4)	0.423
	No	25 (100.0)	46 (100.0)	212 (98.6)	
Any liver-directed therapy	Yes	12 (48.0)	20 (43.5)	137 (63.7)	0.020
	No	13 (52.0)	26 (56.5)	78 (36.3)	

Abbreviations: HCC: hepatocellular carcinoma.

**Table 4:** Multivariate analysis of predictors of overall survival.

		Hazard Ratio	95% CI	*p*-Value
ECOG performance status	0–1	0.417	0.300–0.579	<0.001
	≥2	1	REF	
Alpha-fetoprotein (µg/L)	<400	0.679	0.527–0.873	0.003
	≥400	1	REF	
Quaternary versus non-quaternary treatment center	Quaternary	0.652	0.503–0.844	0.001
	Non-quaternary	1	REF	

Abbreviations: ECOG: Eastern Cooperative Oncology Group. CI: confidence interval. Adjusted for age, gender, race, Child–Pugh status, metastatic disease at diagnosis, and population center size.
